# Personal Health Data in Healthcare: Important Factors Considered by Health Students—A Qualitative Study

**DOI:** 10.3390/healthcare14121731

**Published:** 2026-06-16

**Authors:** Sjors W. M. Groeneveld, Gaya Bin Noon, Mathieu Figeys, Lisette van Gemert-Pijnen, Rudolf M. Verdaasdonk, Plinio Pelegrini Morita, Shaniff Esmail, Harmieke van Os-Medendorp, Marjolein E. M. den Ouden

**Affiliations:** 1 Research Group Technology, Health & Care, School of Social Work, Saxion University of Applied Sciences, 7513 AB Enschede, The Netherlands; 2 TechMed Centre, Health Technology Implementation, University of Twente, 7522 NB Enschede, The Netherlands; 3 School of Public Health Sciences, University of Waterloo, Waterloo, ON N2L 3G1, Canada; 4 School of Nursing and Midwifery, University of Newcastle, Callaghan, NSW 2308, Australia; 5 Centre for eHealth and Wellbeing Research, Section of Psychology, Health and Technology, University of Twente, 7522 NB Enschede, The Netherlands; 6 Research Institute for Aging, University of Waterloo, Waterloo, ON N2L 3G1, Canada; 7 Department of Systems Design Engineering, University of Waterloo, Waterloo, ON N2L 3G1, Canada; 8 Centre for Digital Therapeutics, Techna Institute, University Health Network, Toronto, ON M5G 2C4, Canada; 9 Department of Occupational Therapy, University of Alberta, Edmonton, AB T6G 2G4, Canada; 10 Faculty of Health, Sports and Social Work, Inholland University of Applied Sciences, 1095 PN Amsterdam, The Netherlands; 11 Spaarne Gasthuis Academy, 2000 AK Haarlem, The Netherlands; 12 Research Group Care and Technology, Regional Community College of Twente, 7553 VZ Hengelo, The Netherlands

**Keywords:** personal health data, health professional education, preventive healthcare, digital health, data literacy, healthcare students, artificial intelligence, qualitative research

## Abstract

**Highlights:**

**What are the main findings?**

- Health students shared perspectives on personal health data that clustered around three overarching domains: (1) personalization and prevention, (2) data quality and ethical considerations, and (3) organizational implications and conditions.
- Within these domains, health students described personal health data as a valuable resource for more personalized and preventive care, while emphasizing concerns about data quality, ethical use, and the organizational and professional changes required to support its use.

**What is the implication of the main findings?**

- Preparing future healthcare professionals for data-driven healthcare requires integrating critical data literacy, ethical reflection, and interdisciplinary collaboration into health professional education, while ensuring that organizational conditions support the responsible use of personal health data.

**Abstract:**

**Background/Objectives:** Digital technologies and data-driven approaches are rapidly transforming healthcare practice and enabling more personalized and preventive care. As personal health data becomes increasingly embedded in healthcare systems, understanding how future healthcare professionals interpret these developments is essential for shaping responsive health education. This study aims to identify the factors that students in health-related programs consider important regarding the increasing use of personal health data in healthcare. **Methods:** An exploratory qualitative focus group study was conducted between March 2024 and July 2025 across five higher education institutions in Australia, Canada, and the Netherlands. Seven focus groups were conducted with forty students from health-related programs, including nursing, public health, occupational therapy, and social work. Participants discussed the use of personal health data in healthcare and reflected on short fictional future scenarios designed to stimulate discussion about possible developments in data-driven healthcare. Data were analyzed using reflexive thematic analysis using ATLAS.ti. **Results:** Three overarching domains were identified: (1) personalization and prevention, (2) data quality and ethical considerations, and (3) organizational implications and conditions. Students described personal health data as a powerful tool for personalization, prevention, and informed decision-making. At the same time, they raised concerns about data reliability, overreliance on automated systems, patient anxiety, potential dehumanization of care, privacy risks, and emerging inequalities related to access to and representation within data systems. Overall, students appeared neither purely techno-optimistic nor technophobic, but articulated nuanced ethical, cultural, and professional tensions surrounding data-driven care. **Conclusions:** Preparing future healthcare professionals for data-driven healthcare requires integrating critical data literacy, ethical reflection, interdisciplinary collaboration and opportunities to critically engage with the societal and professional implications of data-driven technologies into health professional education, while ensuring that organizational conditions support the responsible use of personal health data.

## 1. Introduction

In recent years, healthcare systems have seen a noticeable increase in the collection and use of care recipients’ personal health data [1,2,3]. In this context, personal health data refers to real-world patient data that can be used to inform personalized treatments, interventions, and research [4]. Such data may include medical information, lifestyle monitoring data, and social or contextual data collected across clinical, community, and home settings [1,5,6]. Through digital health infrastructures and patient portals, these data are increasingly integrated into healthcare practice to support more personalized, preventive, and population-oriented approaches to care [2,7,8,9].

As the collection and use of personal health data increasingly shape the healthcare landscape, it is essential to understand the perspectives of healthcare professionals who not only integrate data-driven insights into their daily practice, but also actively contribute to the generation and interpretation of these data. First, the growing adoption of data-driven tools is likely to alter job design [10] and work routines [11], requiring sufficient training [12,13,14] to prevent confusion and unnecessary strain such as increased workload [15]. Second, the use of care recipients’ data may conflict with values and ethics central to healthcare professionals, for example, through risks of depersonalization, automated decision-making, limited transparency regarding how data-driven insights are generated or shared [7,12,16], reduced human contact [17], and concerns surrounding privacy and data protection [18]. Finally, understanding professionals’ views is crucial for designing technologies that effectively support real-world clinical demands and practice [19], while redefining roles and responsibilities to fit a data-enriched model of care [20].

When considering the integration of data-driven insights into healthcare practice, it is important to look not only at the current workforce, but also at those who will work in healthcare in the future. Students in health-related programs will become the next generation of professionals, and their views will influence how personal health data is used and understood in the coming years. This includes students in programs such as nursing, social work, public health, and occupational therapy. Understanding their perspectives is therefore not only relevant for anticipating future professional practice, but also for informing the development of health professional education programs that prepare students for data-driven and preventive healthcare environments. Although this generation may be familiar with digital technologies in daily life, this does not necessarily translate into acceptance in healthcare contexts, particularly given the “privacy paradox,” where individuals value digital convenience while expressing concerns about privacy [21].

The current literature includes studies on health students’ attitudes toward technology, ranging from its use in education [22,23] to its application in healthcare practice [24,25], as well as specifically focused data-driven technologies [26,27,28,29]. However, limited research has explored how students in professional health programs view the increasing use of personal health data, despite such data forming the foundation of many emerging healthcare technologies and preventive care approaches. This gap leaves uncertainty about how future professionals may respond to data-driven care environments, which is particularly relevant in the context of ongoing discussions about innovation in health professional education and the growing role of digital technologies in preventive healthcare. Understanding these perspectives may therefore help inform how education and training should evolve to prepare future professionals to work responsibly with data-driven tools while supporting prevention-oriented and patient-centered care [12,13]. This study thereby contributes to ongoing discussions regarding digital health literacy, professional identity, and responsible implementation of data-driven healthcare.

Therefore, the aim of this study is to identify the factors that students in health-related programs consider important regarding the increasing use of personal health data in care.

## 2. Materials and Methods

### 2.1. Study Design

Between March 2024 and July 2025, we conducted an open, exploratory qualitative focus group study to identify the factors that students in health-related programs consider important regarding the increased use of personal health data in healthcare.

#### 2.1.1. Study Setting

The study was conducted across five higher education institutions offering health-related programs in Australia, Canada, and the Netherlands. These institutions provide professional training for future healthcare workers in fields such as nursing, health sciences, public health, and social work, and integrate varying levels of digital health and data-related content into their curricula. Three of the five involved institutions collaborate closely through a joint research agenda aimed at educating future-proof healthcare professionals, focusing on innovation, digitalization, and preparing students for emerging data-driven practices. The international and multidisciplinary setting allows for a diverse range of student perspectives on the use of personal health data in healthcare practice.

#### 2.1.2. Study Population

The focus groups included both undergraduate and graduate students enrolled in scientific and professional health-related programs at universities in Australia, Canada, and the Netherlands. Eligible participants were required to be at least 18 years old, and proficient in either English (Australia, Canada) or Dutch (Netherlands). A purposive sampling approach was used to recruit students from different health-related programs and educational contexts in order to capture a broad range of perspectives on the use of personal health data in healthcare. To support this, participants were recruited through multiple channels, including on-campus advertisements (e.g., posters), digital invitations through email and social media, posts on online learning management platforms, and direct communication from faculty members (not moderating the focus groups) who informed students about the opportunity to participate. Participation was voluntary, with no consequences for non-participation and no dependency relationship between researchers and students. Interested students were contacted via email and provided with additional details about the study’s objectives. Those who agreed to participate received an informational letter outlining the research and focus group procedures. Prior to commencing data collection in the focus groups, participants provided signed informed consent. Focus groups were conducted until saturation had been achieved by obtaining sufficient depth and variation in perspectives to address the study aim, with later focus groups primarily contributing additional nuance and confirmation of existing insights rather than substantially new themes.

#### 2.1.3. Ethical Approval

Ethical approval for this study was provided by the Human Ethics Advisory Panel of the University of Newcastle (#H-2025-0024), the University of Waterloo Research Ethics Boards (REB #46039), the University of Alberta Research Ethics Boards (#Pro00140482), and the Ethics Committee of the University of Twente (#240105). This study involving student participants was conducted in accordance with the Australian National Statement on Ethical Conduct in Human Research (Australia); the Tri-Council Policy Statement: Ethical Conduct for Research Involving Humans (TCPS 2) (Canada); and the Dutch Code of Conduct for Research Integrity. All participants provided informed consent.

### 2.2. Data Collection and Analysis

#### 2.2.1. Data Collection

The focus groups were moderated by a graduate researcher (SG) trained in qualitative research methods. The moderator facilitated structured discussions, ensuring all participants were actively engaged while maintaining a neutral role with no prior relationship to the participants. Focus groups were conducted either in person (five sessions) or online (two sessions via Microsoft Teams (Microsoft Corporation, Redmond, WA, USA; available at https://www.microsoft.com/microsoft-teams, accessed 28 March 2024) or Zoom (Zoom Video Communications, San Jose, CA, USA; available at https://www.zoom.com, accessed 18 July 2025)), depending on the institution and logistical feasibility. All sessions were recorded for analysis. The discussions followed a structured topic list, which was designed to guide the conversation and consisted of three main sections:

1. Introduction: The first section was meant to introduce the participants and have a first exploration to align all participants on the topic of the focus group.
2. Discussion on the use of personal health data in healthcare practice: The second section explored issues related to the use of personal health data in healthcare practice. Questions were informed by literature on technology use, professional roles, and the broader environment, providing structure while allowing participants to share their own expectations, concerns, and examples [30,31,32,33]. Participants were asked to reflect on potential areas of use, the added value of personal health data in care, situations in which such data might be beneficial or problematic, and how it could affect their work. Additional questions addressed professional responsibilities, organizational conditions, and broader societal implications of increasing reliance on personal health data.
3. Scenario-based discussion: The final section consisted of a scenario-based discussion to stimulate deeper reflection. Participants were divided into two subgroups, each watching a short speculative fiction film (approximately eight minutes) developed in a previous project [34]. The films presented contrasting future scenarios of personal health data use, one utopian and one dystopian, based on realistic situations such as monitoring vital signs, automated recommendations, and status updates. The films were used as discussion prompts to facilitate reflection on potential future uses of personal health data. The films enabled participants to visualize and critically reflect on potential future uses of personal health data, an approach increasingly used in research exploring speculative and technology-oriented futures [35]. After the viewing, participants rejoined the full group to engage in a discussion, sharing their reactions, concerns, and insights.

#### 2.2.2. Data Analysis

To analyze the focus groups, we employed a reflexive thematic analysis with an inductive approach [36], as this method is suited for capturing diverse perspectives, concerns, and expectations within a group setting [37]. Recordings were transcribed verbatim, and initial codes were then generated iteratively by two independent coders (SG and LB) using ATLAS.ti (version 24.1.1; ATLAS.ti Scientific Software Development GmbH, Berlin, Germany). These codes were subsequently discussed with other members of the research team (MdO and HvO) to explore patterns, similarities, and differences across the data. Through an iterative and interpretive process, related codes were grouped into potential themes, which were continuously refined and discussed within the multidisciplinary research team. These themes were then organized into broader overarching domains based on conceptual relationships and recurring patterns identified across the focus groups [38]. In line with a reflexive thematic analysis approach, interpretive decisions were shaped through analytical discussion and reflection within the multidisciplinary research team rather than through formal coder agreement. The multidisciplinary research team also reflected throughout the analysis process on how their disciplinary perspectives may have shaped data interpretation. A thematic map of the analysis was then developed to visually represent the relationships between themes. The final analysis was compiled into a report, which included a selection of representative participant quotes to illustrate key themes and insights.

## 3. Results

### 3.1. Participants

In this study, 40 participants from five different universities across three countries took part in seven focus groups. The focus groups consisted of, on average, six participants, which is regarded as an adequate group size for focus group discussions [39]. The focus groups were interdisciplinary with students from different health-related programs participating together rather than in separate discipline-specific groups. Most of the participants were studying nursing (62.5%) or public health sciences (17.5%). See Table 1 for an overview of the participants.

### 3.2. Overview of Domains and Themes

**Figure 1:**
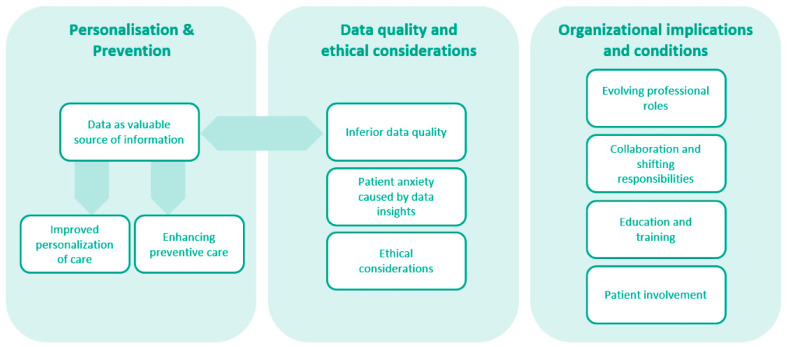
Visual overview of the domains and themes.

The findings of this study are organized into three overarching domains, each consisting of several themes. An overview of all domains and themes is presented in Figure 1, with detailed descriptions provided in Table 2.

#### 3.2.1. Domain 1: Personalization and Prevention

This domain covers three themes: (1) data as valuable source of information, (2) improved personalization of care, and (3) enhancing preventive care. Together, these themes show how students view data as enabling more tailored, proactive, and informed healthcare. Personal health data is first seen as a valuable information source that supports more personalized care and preventive strategies. At the same time, these positive expectations contrast with concerns about data quality and bias discussed in domain two, highlighting a tension between the potential value of data and doubts about its reliability.

##### Data as Valuable Source of Information

Participants described personal health data as a valuable source of information that may support more consistent and evidence-informed decision-making. Data were seen as complementing professional judgment, offering practitioners stronger justification for interventions while giving patients tools for self-awareness and self-advocacy.

“It’s incredibly useful if you can look something back later. […] If something is captured in measurements and numbers, then it’s recorded and you can draw conclusions from it.”

Focus group 1

Continuous monitoring was also frequently described as an important advantage, particularly in contexts where direct observation is limited. Participants emphasized that such data could provide early signals of change, supporting timely intervention, and improving insight into patients’ day-to-day functioning. At the same time, they stressed that data should not be interpreted in isolation but always in relation to clinical assessments and contextual knowledge.

##### Improved Personalization of Care

Building on the previous theme, participants described personal health data as a key enabler of more personalized care by aligning interventions with individual lifestyles and health patterns. Wearables and monitoring tools can reveal patient-centered needs, support early detection, and guide tailored interventions that improve prognosis.

“I think it can also help us personalize and individualize care for that person based on their data.”

Focus group 4

Participants further emphasized that access to longitudinal data can support greater patient involvement in care. Insights into emerging risks and personal trends were described as enabling individuals to adjust their behavior and take a more active role in managing their health. Participants also highlighted the value of pattern recognition at both individual and population levels, where aggregated data may inform predictive models, public health strategies, and efforts to reduce hospital admissions.

##### Enhancing Preventive Care

As a final theme within this domain, participants described personal health data as a powerful tool for supporting preventive care. Continuous monitoring and pattern recognition were seen as enabling the early detection of subtle changes in health status, allowing timely intervention and preventing worsening conditions.

“If you notice a downward trend, you can act on it before something serious happens. […] When you see that decline early, you can step in and say, ‘Okay, let’s start some preventative rehab.’”

Focus group 4

Apps and wearables were also seen as tools that can motivate healthier behavior by tracking progress and supporting self-management. At the same time, participants noted that frequent reminders or prescriptive feedback (e.g., prompts indicating that one “still needs to walk more”) may feel controlling, as the technology may steer behavior rather than support autonomous decision-making.

Participants also highlighted the role of data in monitoring recovery and rehabilitation, as well as identifying broader population-level trends. Aggregated data were seen as enabling the identification of broader trends and informing preventive strategies, contributing to a more proactive understanding of long-term health trajectories.

#### 3.2.2. Domain 2: Data Quality and Ethical Considerations

This domain covers three themes: (1) inferior data quality, (2) patient anxiety caused by data insights, and (3) ethical considerations, highlighting students’ concerns about the risks and unintended consequences of data-driven healthcare.

##### Inferior Data Quality

In contrast to the perceived value of personal health data described in domain one, participants also expressed concerns about the reliability and representativeness of these data. They questioned the accuracy of the data, pointing to potential sensor errors, inconsistent measurements, and the risk of overinterpreting minor changes. Many emphasized that such data should be double-checked and used as supportive information rather than the primary basis for clinical decisions.

“There might be instances where your device isn’t accurate compared to a physician’s assessment. So I think there’s a blurred line between relying on it too much and using it simply as a supplement to your health and lifestyle.”

Focus group 6

Beyond technical limitations, participants also highlighted concerns related to data representativeness. They noted that reliance on personal health data from apps and wearable technologies may introduce bias by excluding individuals who do not or cannot use these tools. This was seen as potentially leading to incomplete or skewed datasets that fail to capture the diversity of populations, thereby reinforcing dominant norms of health and overlooking vulnerable groups.

Participants further reflected on how personal health data can unintentionally create unfair assumptions. Using old records or labels can lead to quick judgments, even when a person’s situation has changed. They worried this could result in unfair treatment or narrow ideas of what is “healthy,” ignoring personal experiences and diverse lifestyles that do not fit standard data patterns.

##### Patient Anxiety Caused by Data Insights

Building on concerns about data quality, participants also described that continuous health monitoring may also create fear, stress, or even hypochondria. Wearable data may lead users to overinterpret minor irregularities, potentially causing panic. Participants noted that additional information may become overwhelming for individuals who lack the expertise to interpret these data appropriately. They also warned that such data might encourage self-diagnosis through online searches, increasing anxiety, and attention to incorrect health problems.

“For individuals, knowing too much can actually create anxiety. If they see something irregular in their heart rate, they might panic or become overly worried about it.”

Focus group 3

In addition, participants warned that this process can result in cognitive overload, obsession with numbers, and a loss of perspective with patients. In this context, more data was not necessarily perceived as beneficial, as excessive detail may reduce trust, increase insecurity, and create additional demands for both patients and professionals.

##### Ethical Considerations

Participants raised a range of ethical concerns related to the increasing use of personal health data in healthcare. A central concern was the potential dehumanization of care, as reliance on data-driven insights may reduce individuals to quantifiable metrics and weaken human interaction. Participants emphasized that data should be interpreted alongside lived experiences, clinical judgment, and contextual understanding, rather than replacing them.

“You don’t want to reduce people to just numbers. You actually want to spend time with the clients.”

Focus group 4

Concerns about privacy and data governance were also prominent. Participants questioned who owns personal health data, who has access to it, and how securely it is managed. The need for transparent policies, meaningful consent, and the ability to control or remove personal health data was frequently emphasized, particularly in relation to the long-term implications of stored health information.

Beyond individual-level concerns, participants highlighted broader societal implications of data use. They noted that unequal access to digital technologies may lead to exclusion from data-driven healthcare, reinforcing existing inequalities. At the same time, concerns were raised about the potential misuse of data by commercial or governmental actors, for example through commercialization or forms of behavioral influence.

“I see the control of health information ending up in the hands of people with conflicting interests, where profit becomes the priority. That data can then be used to influence society, pushing certain practices, shaping behavior, or enabling targeted advertising and that’s definitely something I’m concerned about.”

Focus group 6

Finally, participants reflected on how data-driven systems may embed dominant cultural perspectives in defining what counts as “normal” or “healthy.” This was seen as potentially reinforcing narrow, individualistic understandings of health while overlooking cultural diversity and alternative perspectives.

#### 3.2.3. Domain 3. Organizational Implications and Conditions

This domain covers four themes: (1) evolving roles, (2) collaboration and shifting responsibilities, (3) education and training, and (4) patient involvement, showing how data use reshapes tasks, teamwork, and expectations within healthcare.

##### Evolving Roles

Participants described how the increasing use of personal health data may reshape professional roles in healthcare, shifting them toward a more coaching and interpretive function supporting patients in interpreting their own information. While this empowers individuals, it also raises concerns about trust, responsibility, and professional boundaries. They emphasized that clear guidance, standards, and gradual adaptation are needed to ensure the evolution of this role benefits both patients and professionals.

“I think that the coaching role will become much more prominent than it is now, especially if you assume that people can report things themselves. In that sense, your professional role will definitely change.”

Focus group 1

Participants further distinguished between working with personal health data and analyzing it. While they emphasized the importance of being able to interpret data in clinical contexts, most felt that healthcare professionals should focus on patient care, using data insights rather than processing raw data themselves. Instead, they anticipated a growing role for specialists, such as clinical informaticians or data scientists, who can translate complex datasets into meaningful insights for practice.

At the same time, participants stressed that data-driven systems should support, rather than replace, professional judgment. Clinical decision-making was consistently described as a human responsibility, requiring contextual understanding that cannot be captured by data alone. This reflects a clear boundary in which data informs, but does not determine, care decisions.

“It would be more appropriate if the system simply collected the data and showed trends over time, and then the nurse interpreted that information rather than the system making decisions itself.”

Focus group 7

Finally, participants expressed mixed expectations regarding the impact of data-driven technologies on workload and autonomy. While some anticipated increased efficiency, others expected additional burdens related to data interpretation and system use. More fundamentally, participants raised concerns that constant monitoring and system-driven decisions could reduce personal autonomy for both professionals and patients, highlighting tension between guidance and control in data-driven care.

##### Collaboration and Shifting Responsibilities

Participants emphasized that the effective use of personal health data depends on coordinated teamwork and shared responsibility across healthcare teams. Uneven engagement within teams may lead to inconsistent data use and interpretation, reducing the overall value of the technology. In this context, clear protocols, shared agreements, and open communication were seen as essential conditions for successful implementation.

“I also think it becomes a disadvantage if you’re the only one who wants to use it. Everyone has to agree to it, otherwise the whole system won’t work. If one person records everything and another doesn’t, then nothing is accurate anymore.”

Focus group 2

Participants further highlighted the importance of accurate documentation and information sharing across disciplines and organizations. Fragmented or inconsistent data entry was seen as undermining both the quality of data and its usefulness for clinical decision-making. At the same time, digital systems were described as offering opportunities to strengthen multidisciplinary collaboration, provided that data are consistently and meaningfully recorded.

Finally, participants pointed to the influence of organizational culture on the adoption of data-driven technologies. Individual enthusiasm may fade when the broader team is resistant. Broad support, shared training, and mutual trust were therefore seen as important for sustainable adoption.

##### Education and Training

Participants emphasized the need for structured education and continuous training to prepare healthcare professionals for working with personal health data. Integrating data literacy into curricula was seen as essential, including practical skills in data interpretation as well as critical reflection on privacy, bias, and responsible data use. While some participants reported limited exposure to these topics, others described early experiences during their education, highlighting variation in current training approaches. Participants also emphasized the importance of interdisciplinary education that brings together health, technology, and informatics, reflecting the increasingly collaborative nature of data-driven care.

“I think it’s really important that this is included in our education. I haven’t had much of it yet, and I believe it could be very valuable. […] f you can bring that knowledge into an organization as an intern or new employee, it would be incredibly valuable.”

Focus group 2

In addition, it is seen as important to also address the risk of relying too heavily on personal health data, which could erode professional skills and critical thinking. If systems consistently provide answers, there is a risk of passively accepting outputs rather than questioning them. This over-reliance may reduce health literacy and practical expertise, leaving professionals less capable of independent judgment.

“AI is quite effective. It keeps doing things right, right over and over and over and over again. Eventually it is really easy to just slip up and trust the AI [to] make the decision.”

Focus group 3

##### Patient Involvement

As a final theme, participants emphasized the importance of actively involving patients in understanding and using their personal health data. However, this was seen as dependent on patients’ digital and health literacy. Participants noted that many individuals may lack the skills needed to interpret data appropriately, particularly older adults and those with limited digital competencies. To prevent exclusion, systems should be inclusive, accessible, and supported by clear explanations and guidance.

“Not everyone is able to read or interpret that data, and with an aging population, many older adults today are not able to use or understand these kinds of tools.”

Focus group 1

Finally, participants stressed that informed consent must be meaningful rather than a simple “tick-the-box” step. Patients should retain autonomy over how their data is used, with special attention to vulnerable groups such as children, older adults, and disadvantaged populations to protect them from misinterpretation, misinformation, or exploitation.

## 4. Discussion

### 4.1. Main Findings

The aim of this study was to identify the factors that students in health-related programs consider important regarding the increasing use of personal health data in patient care. We found three overarching domains, each consisting of several themes: (1) personalization and prevention, (2) data quality and ethical considerations, and (3) organizational implications and conditions.

#### 4.1.1. Balancing Opportunities and Concerns in Data-Driven Healthcare

The insight that personal health data can improve personalized care and enhance preventive care is not new, and our findings in this regard align with earlier studies [40,41,42,43]. What stands out, however, is that students described personal health data as a valuable source of information that can support decision-making, reduce bias, and strengthen professional judgment, while at the same time expressing strong doubts about the quality of these data. They mentioned risks such as technical errors, inconsistent measurements, and biased insights resulting from unequal access or outdated records. This suggests that although students are eager to use data, they may currently lack sufficient trust to rely on such systems, even though trust is recognized as an important precondition for the successful adoption of data-driven technologies [44]. In this context, students emphasized the importance of interpreting data within its clinical and social context rather than relying on data alone.

Another notable finding concerns the potential unintended consequences of increased access to personal health data for patients themselves. Students worry that too much insight can become overwhelming, and that constant confrontation with one’s own data may lead to anxiety, obsession, or information overload, ultimately reducing trust in technology. Students also fear that individuals might start self-diagnosing based on their personal health data and online searches, which could increase stress and lead people to focus on incorrect health problems [45]. Beyond these potential psychological effects for patients, students also reflected on broader ethical and societal implications of increasing reliance on personal health data in healthcare.

#### 4.1.2. Ethical, Societal, and Professional Tensions

Within the theme of ethical considerations, students raised several issues that are well known from earlier studies, such as risks of dehumanization, concerns about privacy, and the possibility of social isolation through intensive digitalization [16,18,46,47]. These points show that students are not blind to the potential negative impact of a data-driven future in healthcare. However, beyond these commonly discussed concerns, students also reflected on how data-driven systems may shape definitions of ‘normality’, reinforce dominant perspectives on health, and exclude populations that are insufficiently represented within datasets. Students questioned who ultimately defines what counts as “normal” or “healthy” when such definitions are embedded in algorithms and data infrastructures. They expressed concern that those designing and governing these systems may shape standards of health and behavior in ways that are not transparent to patients or professionals [48]. These perspectives highlight the importance of culturally sensitive approaches to data use in healthcare, as there is a risk of unintentionally excluding or misrepresenting certain populations. They also point to the need to actively involve healthcare professionals in the design and development of data-driven systems and algorithms, in order to enhance transparency, contextual relevance, and accountability.

Beyond ethical concerns, students also raised questions about inequality and access to data-driven healthcare systems. They introduced concerns about how data use may create new forms of inequality due to unequal access to technology and personal data. In the context of data-driven systems, this inequality works in two directions: when certain groups lack access to the technologies that display or generate data, they cannot benefit from the insights produced. At the same time, the absence of their data means that new data systems cannot learn from these groups, which may lead to biased insights and skewed conclusions. In this sense, exclusion from data-driven healthcare is not only a matter of unequal access to technologies, but also of underrepresentation in the datasets that increasingly shape healthcare knowledge, services, and decision-making. For example, previous studies have raised concerns that AI-driven healthcare models trained on non-representative datasets may be less accurate for certain population groups [49,50]. This resonates with the digital divide theory, which describes how inequalities in access to, use of, and benefits from digital technologies can reinforce existing social disparities [51]. In the context of digital health technologies, such inequalities may disproportionately affect already vulnerable populations, including migrants and refugees, due to barriers related to digital literacy, language, access to devices or connectivity, and concerns regarding privacy, trust, and consent [52]. Students described situations in which personal data are collected but not made accessible to the individual, or where such data could be used for inappropriate purposes by commercial companies or government bodies. This reflects broader concerns regarding data ownership, consent, and the potential misuse of personal health information [53].

#### 4.1.3. Implications for Professional Roles and Education

Beyond these broader ethical and societal concerns, students also reflected on how the increasing use of personal health data may reshape professional roles in healthcare. Students expect that they should be able to use data insights in their interactions with patients, but they do not see themselves becoming data specialists. Instead, they point to emerging roles such as clinical informaticians or data scientists who may take responsibility for processing and interpreting complex datasets, reflecting an ongoing debate in the literature about whether such responsibilities should lie with specialized professionals or remain part of the broader clinical role [14]. Although nursing students formed the majority of the study participants, and such coaching and interpretive roles are traditionally more central to nursing practice than to some other represented disciplines, students believe that their professional roles will shift toward a greater coaching and consulting-oriented function as patients will need support in interpreting these data correctly.

These collective findings highlight a broad need for innovation in health professional education, where curricula move beyond traditional clinical competencies to include digital and data literacy, ethical reflection, and interdisciplinary collaboration. This aligns with a substantial body of literature advocating for stronger integration of digital and data competencies in health education [14,54,55,56]. At the same time, students feared that relying too heavily on data systems could undermine professional skills and critical thinking. This concern resembles the concept of skill erosion, in which overdependence on automated systems may lead to a decline in human expertise [57]. For instance, a recent study observed changes in adenoma detection rates during standard, non–AI-assisted colonoscopy after prior exposure to a data-driven AI system, suggesting that continued use of such systems may influence endoscopists’ clinical behavior [58]. These findings suggest that education should not approach data interpretation as a purely technical task. If students come to view automated systems as a ‘safety net’ rather than a tool, there is a risk that foundational clinical reasoning becomes less prominent, particularly in situations where technology fails or data are ambiguous. This highlights the importance of educational approaches that strengthen professionals’ ability to critically interpret and contextualize algorithmic outputs. Consequently, integrating data-related competencies into education in a careful and meaningful way that enables the potential of data-driven care without diminishing the unique, complementary strengths that healthcare professionals bring to clinical practice is considered crucial [6]. For healthcare organizations, this also implies that implementing data-driven systems is not solely a technical or organizational process, but may require ongoing dialog regarding responsibility, trust, interpretation, and the ethical use of personal health data in everyday practice.

Although this study was exploratory and not explicitly guided by a predefined theoretical model, the findings can be related to established frameworks on technology acceptance and implementation, such as UTAUT and the NASSS framework [30,32]. For example, students’ reflections on the value of data, organizational conditions, and evolving professional roles reflect themes that are also described in these frameworks. At the same time, students highlighted aspects that extend beyond the primary focus of these frameworks, including emotional burden, anxiety related to health data, and concerns about the erosion of professional skills, thereby adding a more experiential dimension to existing models. These insights suggest that future healthcare professionals may experience the impact of working with personal health data not only as a question of usefulness or organizational fit, but also as a matter of professional identity and responsibility.

Taken together, these findings highlight the need to carefully integrate personal health data into healthcare practice and education in ways that support preventive care, safeguard ethical principles, and prepare future professionals to work critically and collaboratively with data-driven technologies.

### 4.2. Recommendations for Health Education, Practice, and Further Research

Based on the findings of this study, four recommendations can be made for health education, practice, and further research.

1. Integrate critical data literacy in health professional education
  Students indicated that data-driven healthcare will increasingly shape their future professional roles, yet many reported limited structured training in interpreting health data. Curricula should therefore integrate data literacy as a core competency, including basic data interpretation as well as reflection on privacy, bias, and responsible data use. At the same time, educational approaches should deliberately strengthen interpretive, reflective, and critical competencies to prevent overreliance on automated outputs and support independent professional judgment.
2. Promote multidisciplinary collaboration in data-driven healthcare systems
  Students did not see themselves as primary data analysts but emphasized the importance of collaboration with data specialists such as clinical informaticians and data scientists. This highlights the need for multidisciplinary teams in which technical and clinical expertise complement each other, particularly in the interpretation of data, the translation of insights into practice, and clinical decision-making. Educational programs and healthcare organizations should therefore encourage interdisciplinary collaboration.
3. Ensure equitable and culturally sensitive approaches to personal health data use
  Students raised concerns about unequal access to data-driven systems and the potential exclusion of certain populations from it. Data-driven systems should therefore be designed to be accessible, inclusive, and sensitive to different cultural perspectives on health, privacy, and data ownership, helping to prevent the reinforcement of existing health inequalities.
4. Strengthen research on competencies and co-design in data-driven healthcare
  Further research is needed to identify the core competencies required for different healthcare professionals working with personal health data, and how these may vary across disciplines and contexts. In addition, research should explore co-design approaches that actively involve healthcare professionals, patients, and technical experts in the development of data-driven systems, to ensure alignment with clinical practice, ethical considerations, and real-world needs.
5. Explore cross-cultural and health system differences in data-driven healthcare
  Although this study included participants from multiple countries, it was not designed to compare perspectives across cultural or healthcare system contexts. Future research could therefore explicitly examine how cultural backgrounds and differences in healthcare systems shape perceptions of and engagement with personal health data.

### 4.3. Strengths and Limitations

This study deliberately focused on students as future healthcare professionals who will increasingly work with data-driven technologies. Their perspectives may help inform future professional roles and educational development. Although many students gain experience through internships or part-time work, they typically lack long-term professional experience. Their reflections should therefore be seen as anticipations of future practice rather than insights grounded in extensive clinical experience. In addition, nursing students formed the majority of participants, which may have influenced the emphasis on certain professional values and responsibilities throughout the findings. Furthermore, as participation was voluntary, students with a stronger interest in digital health or data-driven care may have been more likely to participate.

From a methodological perspective, it is worthwhile to mention the use of the two speculative fiction films during the focus groups. Such narrative scenarios can make complex technological futures more tangible and stimulate discussion about opportunities and risks that participants may not yet have encountered in practice [35]. At the same time, the combination of online and in-person focus groups may have influenced group dynamics and interaction patterns across sessions.

The inclusion of students from different countries allowed for a broader range of perspectives. Although the study was not designed as a cross-country comparison, differences in healthcare systems, educational approaches, and the integration of digital technologies across Australia, Canada, and the Netherlands *may* have influenced how students reflected on the role of personal health data in healthcare practice. For example, variations in the implementation of electronic health records, digital health policies, and the extent to which data-driven care is embedded within educational programs may have shaped students’ familiarity with and expectations of these technologies, noting that the Australian representation was relatively limited compared to the Canadian and Dutch participants. In addition, as with all qualitative research, the interpretation of findings is shaped by the analytical perspective of the research team. The interdisciplinary background of the researchers, including perspectives from nursing, health technology, and healthcare research, supported a broad interpretation of the data but may also have influenced the identification and prioritization of themes. To enhance transparency and credibility, illustrative quotes were included. The findings are therefore not intended to be statistically generalizable but may offer insights that are transferable to similar educational and healthcare contexts.

Finally, it is important to acknowledge the rapid development and increasing adoption of data use in healthcare (amongst others in AI systems), during and after the period of data collection. These developments may have already influenced or further shaped the perspectives described in this study. While some of the findings may have been confirmed or reinforced by these advancements, others may evolve as technologies become more embedded in practice. The findings should therefore be interpreted as a reflection of perspectives at a particular moment in an ongoing and rapidly changing field.

## 5. Conclusions

This study explored what students in health-related programs consider important regarding the increasing use of personal health data in patient care. The findings show that future healthcare professionals recognize the potential of personal health data to support more personalized and preventive care, while also expressing concerns about data quality, ethical implications, and unequal access to digital technologies. Rather than adopting purely techno-optimistic or technophobic positions, students articulated nuanced tensions between the opportunities of data-driven healthcare and the need to preserve professional judgment, human interaction, and the ability to interpret data within its broader clinical and social context.

These insights suggest that preparing future healthcare professionals for data-driven healthcare requires more than technical training alone. Future-oriented health professional education should integrate critical data literacy, ethical reflection, interdisciplinary collaboration, and opportunities for students to critically engage with the societal and professional implications of data-driven technologies. At the same time, healthcare organizations and policymakers should recognize that integration of data-driven technologies into healthcare practice depends not only on technological innovation, but also requires ongoing dialog about the role of data in healthcare. By addressing these challenges proactively, health professional education and healthcare systems may better prepare future professionals for increasingly data-driven care environments while ensuring that care remains fair, accessible, and centered on patients.

## Figures and Tables

**Table 1:** Participant Demographics.

Characteristics	Participants (*n* = 40)	
Gender n (%)		
Male	9	(22.5)
Female	31	(77.5)
Mean Age (SD)	25.3	(6.8)
Program of study n (%)		
Nursing	25	(62.5)
Public health sciences	7	(17.5)
Occupational therapy	3	(7.5)
Social work	3	(7.5)
Rehabilitation medicine(research)	1	(2.5)
Health Science and policy	1	(2.5)
Country of study n (%)		
Australia	5	(12.5)
Canada	14	(35.0)
The Netherlands	21	(52.5)

**Table 2:** Domains and themes including definitions.

Domain/Theme	Definition	
1. Personalization and Prevention		
Data as valuable source of information	Personal health data are perceived as a valuable source of information that supports more consistent and evidence-informed decision-making. Data can reduce reliance on subjective judgment, but require interpretation in relation to clinical context, highlighting the importance of data quality and reliability.	
Improved personalization of care	Personal health data enables more personalized care by aligning interventions with individual lifestyles and health patterns. Pattern recognition supports early detection and tailored interventions, while combining individual and population-level data may improve long-term health outcomes.	
Enhancing preventive care	Personal health data supports preventive care through continuous monitoring and early identification of risks. While digital tools can encourage healthier behavior and self-management, tensions arise between support and control, particularly when feedback becomes intrusive or directive.	
2. Data quality and ethical considerations		
Inferior data quality	Concerns about data reliability include technical errors, inconsistent measurements, and limited representativeness due to unequal access across population groups. These issues highlight a tension between the perceived objectivity of data and the risk of bias, misinterpretation, and incomplete insights.	
Patient anxiety caused by data insights	Continuous access to personal health data may lead to increased anxiety, cognitive overload, and overinterpretation of minor changes. This reflects a tension between the availability of detailed information and individuals’ capacity to interpret it meaningfully.	
Ethical considerations	The use of personal health data raises ethical concerns related to dehumanization, privacy, data ownership, and unequal access. In addition, data-driven systems may embed dominant cultural perspectives, shaping definitions of health and potentially reinforcing existing inequalities.	
3. Organizational implications and conditions		
Evolving professional roles	Personal health data contributes to a shift in professional roles toward interpretation and patient support. While data inform decision-making, professional judgment remains central, reflecting a boundary between technological guidance and clinical responsibility.	
Collaboration and shifting responsibilities	Data-driven care requires coordinated teamwork, consistent data practices, and clear division of responsibilities. Effective implementation depends on alignment across teams and organizations, supported by shared protocols, trust, and communication.	
Education and training	Preparing professionals for data-driven care requires structured education in data literacy, ethical reflection, and interdisciplinary collaboration. At the same time, attention is needed to prevent overreliance on data-driven systems and maintain critical thinking and professional expertise.	
Patient involvement	Meaningful patient involvement depends on individuals’ ability to understand and use personal health data, which varies across populations. Ensuring inclusivity, accessibility, and ongoing consent processes is essential to support autonomy and prevent exclusion.	

## Data Availability

The qualitative data generated and analyzed during this study are not publicly available due to ethical and privacy restrictions related to the focus group discussions. Anonymized excerpts supporting the findings are included in the article. Additional information about the study procedures may be available from the corresponding author upon reasonable request. The short films used as discussion stimuli were developed in a previous project and are not publicly available due to copyright restrictions.

## Abbreviations

The following abbreviations are used in this manuscript:

AI	Artificial Intelligence
NASSS	Nonadoption, Abandonment, Scale-up, Spread, and Sustainability
UTAUT	Unified Theory of Acceptance and Use of Technology
REB	Research Ethics Board
TCPS 2	Tri-Council Policy Statement: Ethical Conduct for Research Involving Humans

## References

[1] Esmaeilzadeh P. (2024). Challenges and strategies for wide-scale artificial intelligence (AI) deployment in healthcare practices: A perspective for healthcare organizations. Artif. Intell. Med..

[2] Alowais S.A., Alghamdi S.S., Alsuhebany N., Alqahtani T., Alshaya A.I., Almohareb S.N., Aldairem A., Alrashed M., Bin Saleh K., Badreldin H.A. (2023). Revolutionizing healthcare: The role of artificial intelligence in clinical practice. BMC Med. Educ..

[3] Shang Z. (2021). A Concept Analysis on the Use of Artificial Intelligence in Nursing. Cureus.

[4] Alam P., Bolio A., Lin L., Larson H.J. (2024). Stakeholders’ perceptions of personal health data sharing: A scoping review. PLoS Digit. Health.

[5] Masawi T.J., Miller E., Rees D., Thomas R. (2025). Clinical perspectives on AI integration: Assessing readiness and training needs among healthcare practitioners. J. Decis. Syst..

[6] Groeneveld S., Bin Noon G., den Ouden M.E., van Os-Medendorp H., van Gemert-Pijnen J., Verdaasdonk R.M., Morita P.P. (2024). The Cooperation Between Nurses and a New Digital Colleague “AI-Driven Lifestyle Monitoring” in Long-Term Care for Older Adults. JMIR Nurs..

[7] Shaik T., Tao X., Higgins N., Li L., Gururajan R., Zhou X., Acharya U.R. (2023). Remote patient monitoring using artificial intelligence: Current state, applications, and challenges. WIREs Data Min. Knowl. Discov..

[8] Ait Abdelouahid R., Debauche O., Mahmoudi S., Marzak A. (2023). Literature review: Clinical data interoperability models. Information.

[9] Patel M.S., Volpp K.G., Small D.S., Kanter G.P., Park S.-H., Evans C.N., Polsky D. (2023). Using remotely monitored patient activity patterns after hospital discharge to predict 30 day hospital readmission: A randomized trial. Sci. Rep..

[10] Dermody G., Fritz R. (2019). A conceptual framework for clinicians working with artificial intelligence and health-assistive Smart Homes. Nurs. Inq..

[11] Wrede C., Braakman-Jansen A., van Gemert-Pijnen L. (2021). Requirements for Unobtrusive Monitoring to Support Home-Based Dementia Care: Qualitative Study Among Formal and Informal Caregivers. JMIR Aging.

[12] Taneri G.U. (2020). Artificial Intelligence & Higher Education: Towards Customized Teaching and Learning, and Skills for an AI World of Work.

[13] Tolsgaard M.G., Boscardin C.K., Park Y.S., Cuddy M.M., Sebok-Syer S.S. (2020). The role of data science and machine learning in Health Professions Education: Practical applications, theoretical contributions, and epistemic beliefs. Adv. Health Sci. Educ..

[14] Groeneveld van Os-Medendorp H., van Gemert-Pijnen J.E.W.C., Verdaasdonk R.M., van Houwelingen T., Dekkers T., den Ouden M.E.M. (2025). Essential competencies of nurses working with AI-driven lifestyle monitoring in long-term care: A modified Delphi study. Nurse Educ. Today.

[15] Mohammadnejad F., Freeman S., Klassen-Ross T., Hemingway D., Banner D. (2023). Impacts of Technology Use on the Workload of Registered Nurses: A Scoping Review. J. Rehabil. Assist. Technol. Eng..

[16] Kennedy M.R., Huxtable R., Birchley G., Ives J., Craddock I. (2021). “A Question of Trust” and “a Leap of Faith”-Study Participants’ Perspectives on Consent, Privacy, and Trust in Smart Home Research: Qualitative Study. JMIR mHealth uHealth.

[17] Rubeis G. (2023). Guardians of humanity? The challenges of nursing practice in the digital age. Nurs. Philos..

[18] Tran V.-T., Riveros C., Ravaud P. (2019). Patients’ views of wearable devices and AI in healthcare: Findings from the ComPaRe e-cohort. npj Digit. Med..

[19] Groeneveld S.W.M., Dekkers T., van Gemert-Pijnen J., Verdaasdonk R.M., Verveda T.J., Witteveen R., van Os-Medendorp H., den Ouden M.E.M. (2025). Understanding the Factors that Influence the Implementation of AI-Driven Lifestyle Monitoring in Long-Term Care for Older Adults. Gerontologist.

[20] Booth R.G., Strudwick G., McBride S., O’Connor S., López A.L.S. (2021). How the nursing profession should adapt for a digital future. BMJ.

[21] Norberg P.A., Horne D.R., Horne D.A. (2007). The privacy paradox: Personal information disclosure intentions versus behaviors. J. Consum. Aff..

[22] Terkes N., Celik F., Bektas H. (2019). Determination of nursing students’ attitudes towards the use of technology. Jpn. J. Nurs. Sci..

[23] Kemp A., Palmer E., Strelan P., Thompson H. (2024). Testing a novel extended educational technology acceptance model using student attitudes towards virtual classrooms. Br. J. Educ. Technol..

[24] Wilson C.B., Slade C., Wong W.Y.A., Peacock A. (2020). Health care students experience of using digital technology in patient care: A scoping review of the literature. Nurse Educ. Today.

[25] Dallora A.L., Andersson E.K., Gregory Palm B., Bohman D., Björling G., Marcinowicz L., Stjernberg L., Anderberg P. (2024). Nursing Students’ Attitudes Toward Technology: Multicenter Cross-Sectional Study. JMIR Med. Educ..

[26] Labrague L.J., Aguilar-Rosales R., Yboa B.C., Sabio J.B., de Los Santos J.A. (2023). Student nurses’ attitudes, perceived utilization, and intention to adopt artificial intelligence (AI) technology in nursing practice: A cross-sectional study. Nurse Educ. Pract..

[27] Moldt J.-A., Festl-Wietek T., Madany Mamlouk A., Nieselt K., Fuhl W., Herrmann-Werner A. (2023). Chatbots for future docs: Exploring medical students’ attitudes and knowledge towards artificial intelligence and medical chatbots. Med. Educ. Online.

[28] Mousavi Baigi S.F., Sarbaz M., Ghaddaripouri K., Ghaddaripouri M., Mousavi A.S., Kimiafar K. (2023). Attitudes, knowledge, and skills towards artificial intelligence among healthcare students: A systematic review. Health Sci. Rep..

[29] Parpani M.R.S., Dhende M.N., Gawade M.N., Mahapure M.P., Patil M.S., Patole M.V. (2025). Knowledge and Attitude regarding Artificial Intelligence in Health Care among Nursing Students. Int. J. Pharm. Res. Technol. (IJPRT).

[30] Venkatesh V., Morris M.G., Davis G.B., Davis F.D. (2003). User Acceptance of Information Technology: Toward a Unified View. MIS Q..

[31] Schepman A., Rodway P. (2020). Initial validation of the general attitudes towards Artificial Intelligence Scale. Comput. Hum. Behav. Rep..

[32] Greenhalgh T., Wherton J., Papoutsi C., Lynch J., Hughes G., A’Court C., Hinder S., Fahy N., Procter R., Shaw S. (2017). Beyond Adoption: A New Framework for Theorizing and Evaluating Nonadoption, Abandonment, and Challenges to the Scale-Up, Spread, and Sustainability of Health and Care Technologies. J. Med. Internet Res..

[33] Maag M.M. (2006). Nursing students’ attitudes toward technology: A national study. Nurse Educ..

[34] Groeneveld S.W.M., van Os-Medendorp H., van Gemert-Pijnen J., Verdaasdonk R.M., den Ouden M.E.M. (2025). KEYNOTE. THE DIGITAL DATA DIVIDE: USING SPECULATIVE FICTION TO FACILITATE PUBLIC DIALOGUE ON THE INCREASED USE OF PERSONAL DATA. SOCIETY TECHNOLOGY SOLUTIONS. Proc. Int. Sci. Conf..

[35] Groeneveld S., Wentzel J., Laurens M., van Gemert-Pijnen J., Verdaasdonk R., van Os-Medendorp H., den Ouden M. (2026). The Use of Speculative Fiction in Future Focused Healthcare Research: Viewpoint. J. Med. Internet Res..

[36] Braun V., Clarke V. (2021). Can I use TA? Should I use TA? Should I not use TA? Comparing reflexive thematic analysis and other pattern-based qualitative analytic approaches. Couns. Psychother. Res..

[37] Pyo J., Lee W., Choi E.Y., Jang S.G., Ock M. (2023). Qualitative Research in Healthcare: Necessity and Characteristics. J. Prev. Med. Public Health.

[38] Braun V., Clarke V. (2019). Reflecting on reflexive thematic analysis. Qual. Res. Sport Exerc. Health.

[39] Krueger R.A. (2014). Focus Groups: A Practical Guide for Applied Research.

[40] Islam M.M., Mim S.S. (2023). Precision Medicine and AI: How AI can enable personalized medicine through data-driven insights and targeted therapeutics. Int. J. Recent Innov. Trends Comput. Commun..

[41] Sunny M.N.M., Saki M.B.H., Al Nahian A., Ahmed S.W., Shorif M.N., Atayeva J., Rizvi S.W.A. (2024). Optimizing healthcare outcomes through data-driven predictive modeling. J. Intell. Learn. Syst. Appl..

[42] Adeniran I.A., Efunniyi C.P., Osundare O.S., Abhulimen A.O. (2024). Data-driven decision-making in healthcare: Improving patient outcomes through predictive modeling. Eng. Sci. Technol. J..

[43] Rahman M.H., Uddinb M.K.S., Hossanc K.M.R., Hossaind M.D. (2024). The role of predictive analytics in early disease detection: A data-driven approach to preventive healthcare. J. Learn. Sci..

[44] Duenas-Cid D., Calzati S. (2023). Dis/Trust and data-driven technologies. Internet Policy Rev..

[45] Farnood A., Johnston B., Mair F.S. (2020). A mixed methods systematic review of the effects of patient online self-diagnosing in the ‘smart-phone society’on the healthcare professional-patient relationship and medical authority. BMC Med. Inform. Decis. Mak..

[46] Rubeis G. (2023). Adiaphorisation and the digital nursing gaze: Liquid surveillance in long-term care. Nurs. Philos..

[47] Rubeis G. (2020). The disruptive power of artificial intelligence. Ethical aspects of gerontechnology in elderly care. Arch. Gerontol. Geriatr..

[48] Eke C.I., Shuib L. (2025). The role of explainability and transparency in fostering trust in AI healthcare systems: A systematic literature review, open issues and potential solutions. Neural Comput. Appl..

[49] Kleinberg G., Diaz M.J., Batchu S., Lucke-Wold B. (2022). Racial underrepresentation in dermatological datasets leads to biased machine learning models and inequitable healthcare. J. Biomed Res..

[50] Laws E., Charalambides M., Vadera S., Keller E., Alderman J., Blackboro B., Hogg J., Salisbury T., Palmer J., Calvert M. (2025). Diversity and inclusion within datasets in heart failure: A systematic review. JACC Adv..

[51] Reddy H., Joshi S., Joshi A., Wagh V. (2022). A critical review of global digital divide and the role of technology in healthcare. Cureus.

[52] Matlin S.A., Hanefeld J., Corte-Real A., da Cunha P.R., de Gruchy T., Manji K.N., Netto G., Nunes T., Şanlıer İ., Takian A. (2025). Digital solutions for migrant and refugee health: A framework for analysis and action. Lancet Reg. Health–Eur..

[53] Narkhede M.R., Wankhede N.I., Kamble A.M. (2025). Enhancing patient autonomy in data ownership: Privacy models and consent frameworks for healthcare. J. Digit. Health.

[54] Russell R.G., Novak L.L., Patel M., Garvey K.V., Craig K.J.T., Jackson G.P., Moore D., Miller B.M. (2023). Competencies for the use of artificial intelligence–based tools by health care professionals. Acad. Med..

[55] Civaner M.M., Uncu Y., Bulut F., Chalil E.G., Tatli A. (2022). Artificial intelligence in medical education: A cross-sectional needs assessment. BMC Med. Educ..

[56] Charow R., Jeyakumar T., Younus S., Dolatabadi E., Salhia M., Al-Mouaswas D., Anderson M., Balakumar S., Clare M., Dhalla A. (2021). Artificial Intelligence Education Programs for Health Care Professionals: Scoping Review. JMIR Med. Educ..

[57] Rinta-Kahila T., Penttinen E., Salovaara A., Soliman W., Ruissalo J. (2023). The vicious circles of skill erosion: A case study of cognitive automation. J. Assoc. Inf. Syst. Assoc. Inf. Syst..

[58] Budzyń K., Romańczyk M., Kitala D., Kołodziej P., Bugajski M., Adami H.O., Blom J., Buszkiewicz M., Halvorsen N., Hassan C. (2025). Endoscopist deskilling risk after exposure to artificial intelligence in colonoscopy: A multicentre, observational study. Lancet Gastroenterol. Hepatol..

